# Sclerosing Stromal Tumor of the Ovary Presenting as Meigs Syndrome During Childhood

**DOI:** 10.7759/cureus.31562

**Published:** 2022-11-16

**Authors:** Sana Hatoum, Pascal Jarjoura, Charbel Saade, Lena Naffaa

**Affiliations:** 1 Radiology, American University of Beirut Medical Center, Beirut, LBN; 2 Obstetrics and Gynecology, Cleveland Clinic, Cleveland, USA; 3 CT Technology, University College Cork, Cork, IRL; 4 Radiology, Nemours Children’s Hospital, Orlando, USA

**Keywords:** pelvic mass, pediatric surgery, adolescent gynecology, ca-125 antigen, salpingo-oophorectomy, fertility preservation, pleural effusion, ascites, ovarian neoplasm, meigs syndrome

## Abstract

Meigs syndrome is a rare disorder characterized by a triad of benign ovarian tumor, ascites, and pleural effusion. Despite its benign nature, its presentation can be similar to metastatic malignancy, making preoperative diagnosis often challenging. The differential diagnosis includes serious and even life-threatening conditions. Meigs syndrome is most common in postmenopausal women and is extremely rare in children. It is often undiagnosed until an invasive surgery is performed. The fact that surgery includes a unilateral salpingo-oophorectomy in females of reproductive age raises concerns for future fertility and other risks associated with this intervention. Familiarity of radiologists and pediatric surgeons with clinical and imaging findings is beneficial to improve preoperative planning, thereby minimizing invasive surgery and preserving ovarian tissue.

## Introduction

Meigs syndrome is a rare disorder [1] comprising a triad of benign ovarian tumor, ascites, and pleural effusion. Despite its benign nature, its presentation mimics a metastatic malignancy [2], rendering preoperative diagnosis often challenging. The differential diagnosis includes serious and even life-threatening conditions such as metastatic ovarian cancer, congestive heart failure, liver cirrhosis, tuberculosis, and nephrotic syndrome [3]. Meigs syndrome is most common in postmenopausal women [1], with the peak incidence in the seventh decade [3]. It is extremely rare in children [4].

Despite its benign nature, it is often undiagnosed until an invasive surgery is performed. The surgery typically includes a unilateral salpingo-oophorectomy in females of reproductive age [4]. This raises concerns for future fertility and other risks associated with this intervention. Familiarity of radiologists and pediatric surgeons with clinical and imaging findings is extremely helpful for better preoperative planning to minimize invasive surgery and preserve ovarian tissue.

## Case presentation

A previously healthy premenarchal 12-year-old female presented with acute abdominal pain associated with a three-month history of abdominal distention and unintentional weight loss of 3 kg. Physical examination revealed a shifting dullness suggestive of ascites and a palpable mass in the pelvis.

**Figure 1:**
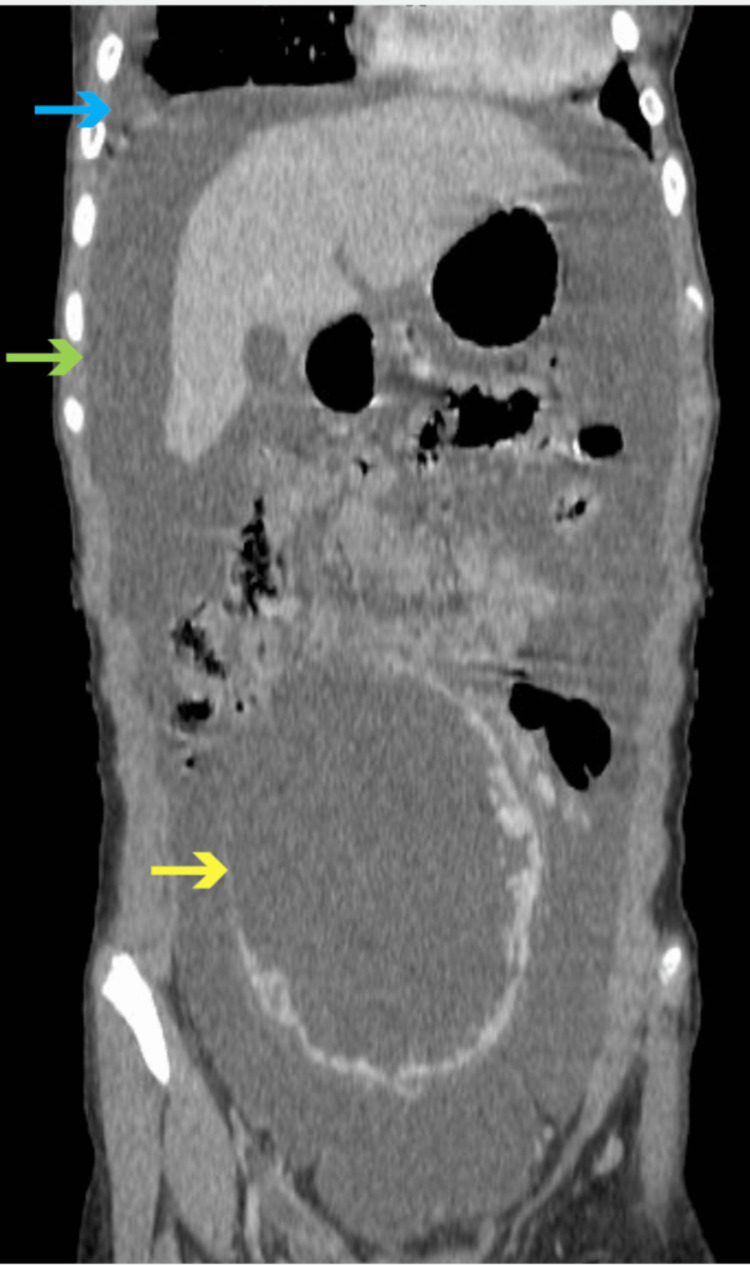
Computed tomography. Mild-to-moderate right pleural effusion (blue arrow) and a large heterogeneously enhancing complex cystic mass arising from the left ovary measuring 14.8 × 12.5 × 6 cm (yellow arrow), along with large ascites (green arrow) were noted.

Laboratory workup showed cancer antigen 125 (CA-125) to be elevated to 1,160 U/mL with normal carcinoembryonic antigen, cancer antigen 19-9, alpha-fetoprotein, beta-human chorionic gonadotropin, and lactate dehydrogenase. Ultrasound revealed a large left adnexal mass measuring 14 × 12 × 6 cm with significant ascites. Computed tomography of the chest, abdomen, and pelvis showed mild-to-moderate right pleural effusion and a large heterogeneously enhancing complex cystic mass arising from the left ovary measuring 14.8 × 12.5 × 6 cm, along with large ascites (Figure 1).

**Figure 2:**
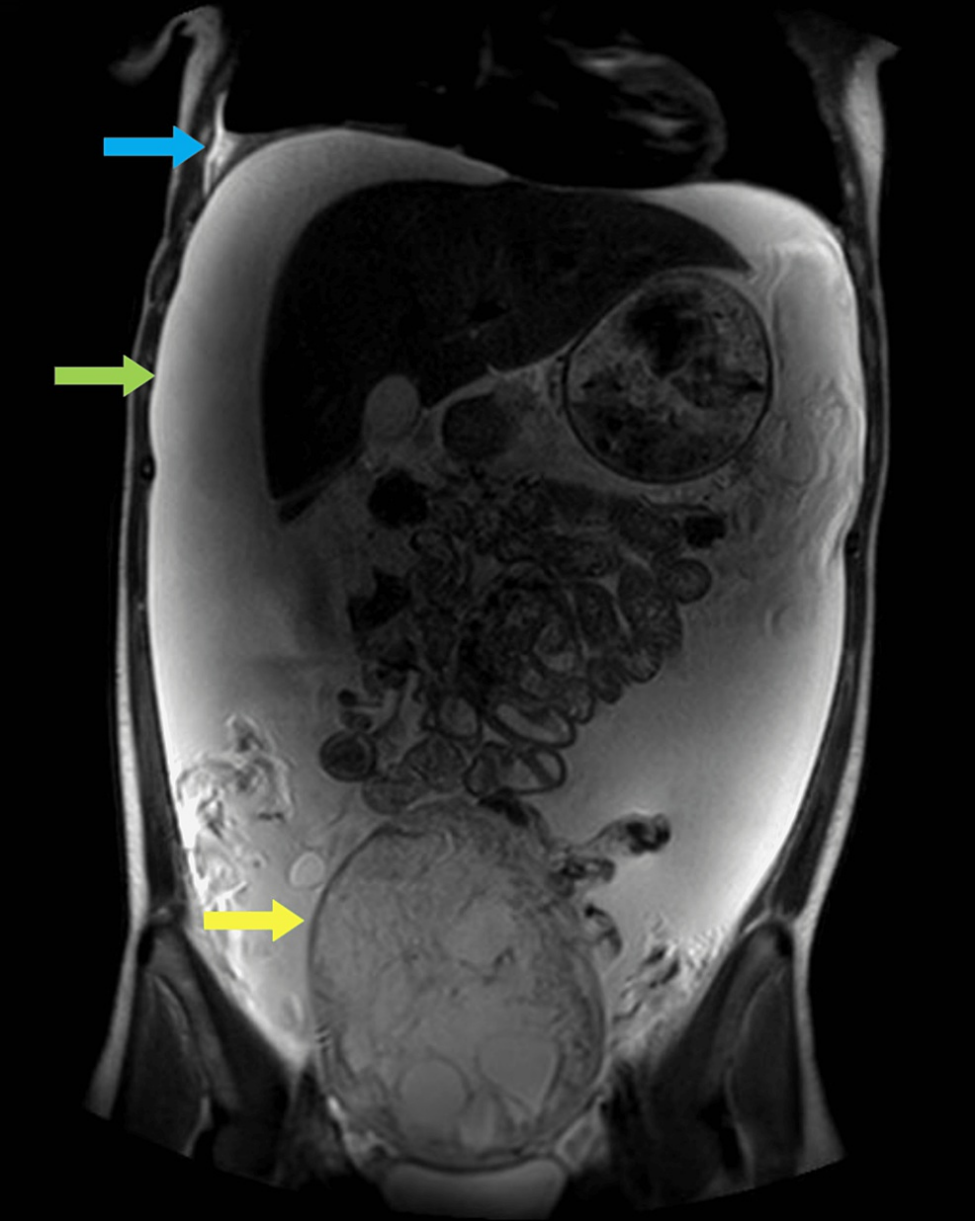
Magnetic resonance imaging with gadolinium. A large 14.6 × 11.8 × 8.4 cm peripherally enhancing complex cystic mass appeared to be arising from the left adnexa and showed internal septations which seemed to be enhancing as well (yellow arrow). The mass was abutting the dome of the bladder with no definite invasion. Secondary large-volume ascites (green arrow) and a moderate right pleural effusion with underlying atelectasis (blue arrow) were noted.

Magnetic resonance imaging of the abdomen and pelvis with intravenous gadolinium was obtained for better local staging redemonstrating the complex cystic mass with internal septations with mild mass effect on the adjacent structures but with no evidence of local invasion or metastatic disease (Figure 2).

A large amount of pleuritic fluid was obtained by ultrasound-guided drainage and was negative for malignant cells. The patient then underwent a fertility-sparing left salpingo-oophorectomy and drainage of the ascitic fluid. Pathology showed a sclerosing stromal tumor of the left ovary. The ascitic fluid was devoid of malignant cells.

Postoperatively, there was a spontaneous resolution of ascites and pleural effusion. The CA-125 level returned to normal.

## Discussion

Meigs syndrome is a rare disease that complicates 1-3% of ovarian fibromas. It represents 2-5% of surgically removed ovarian tumors. Meigs syndrome is most common in postmenopausal women [1], and its incidence peaks in the seventh decade [3]. It can also be seen in children [1] in whom the annual incidence of ovarian masses is only 2.6 cases/100,000 girls [5], making ovarian masses a rare occurrence during childhood and Meigs syndrome even rarer [4].

Its presentation with a triad of ascites, pleural effusion, and ovarian tumor, which is more often seen in more serious conditions, makes it challenging to diagnose Meigs syndrome, a benign disease [6]. Malignant conditions presenting as adnexal masses in pediatric and adolescent patients include germ cell tumors, sex cord-stromal tumors, epithelial ovarian tumors, and metastatic tumors [7]. Although the elevated serum CA-125 is usually suspicious of ovarian cancer, it can also be significantly increased in Meigs syndrome. This increase can be attributed to tumor growth, which increases intra-abdominal pressure and irritates mesothelial cells [8]. The pathophysiology of pleural effusion and ascites has also been discussed in the literature. The direct cause of pleural fluid formation is believed to be the translocation of ascites via diaphragmatic pores [2]. The majority of patients have an exudative pleural effusion, but a transudate pleural effusion cannot rule out Meigs syndrome [2]. Moreover, pleural effusion has been shown to be more commonly right-sided [9], as in the presented case.

The peritoneal fluid formation may be linked to inflammatory cytokines and growth factor release, leading to increased vascular permeability and capillary leakage. Other hypotheses suggest that ascites is attributed to stromal tumor edema and transudation of interstitial fluid. The pathogenesis of ascites may be related to both mechanisms, and the features of both the ascitic fluid and the pleural fluid depend on the relative contribution of each mechanism [2].

The presence of ascites and pleural effusion, along with elevated serum CA-125 and the solid consistency of the tumor, often points toward a malignant disease. Therefore, planning for salpingo-oophorectomy or oophorectomy is common [9]. However, the prognosis is favorable as tumor resection is curative, and the likelihood of recurrence is low [8]. With proper management, life expectancy after surgical removal is similar to that of the general population [3]. In light of a low recurrence rate and extremely good prognosis in cases of ovarian fibroma/fibrothecoma, ovarian-sparing surgery helps preserve reproductive potential and ovarian hormonal function in the long run [9].

The diagnosis and treatment of Meigs syndrome in a female of reproductive age, let alone a child, is extremely challenging. The broad differential diagnosis along with the high suspicion of malignancy imposes an extensive workup and an aggressive intervention. As part of the workup, paracentesis and thoracentesis are often required, but exploratory laparotomy with staging is the treatment of choice. An intraoperative frozen section is usually performed to determine the nature of the tumor and the extent of the surgery. The age of the patient plays a role in the surgical procedure as a patient of reproductive age would undergo unilateral salpingo-oophorectomy, while a postmenopausal woman would undergo total abdominal hysterectomy with bilateral salpingo-oophorectomy [3].

For the pediatric age group, no standard of care is established for Meigs syndrome and ovarian fibromas. However, removing the ovary is not usually required unless the mass cannot be separated from the ovary (like in our case), and conserving both ovaries should be a priority in an expected benign course [4]. Unilateral oophorectomy may negatively affect later oocyte production and patients may experience earlier menopause, which may, in turn, result in negative health outcomes such as earlier cardiovascular disease and osteoporosis [10].

To confirm Meigs syndrome, postoperative resolution of ascites and pleural effusion and histological confirmation of the tumor are usually required [3].

## Conclusions

We present a case of Meigs syndrome in a pediatric patient, a rare condition in a rare age group for such a diagnosis. This benign disease must remain on the differential diagnoses for every female presenting with the classic triad because recognizing Meigs syndrome early in the workup course allows for less aggressive intervention compared to cases of malignancy. The main aim must be preserving ovarian tissue, particularly in the reproductive age group.
